# Beware of Simple Methods for Structure-Based Virtual Screening: The Critical Importance of Broader Comparisons

**DOI:** 10.1021/acs.jcim.3c00218

**Published:** 2023-02-27

**Authors:** Viet-Khoa Tran-Nguyen, Pedro J. Ballester

**Affiliations:** † Centre de Recherche en Cancérologie de Marseille, Marseille 13009, France; ‡ Department of Bioengineering, Imperial College London, London SW7 2AZ, U.K.

## Abstract

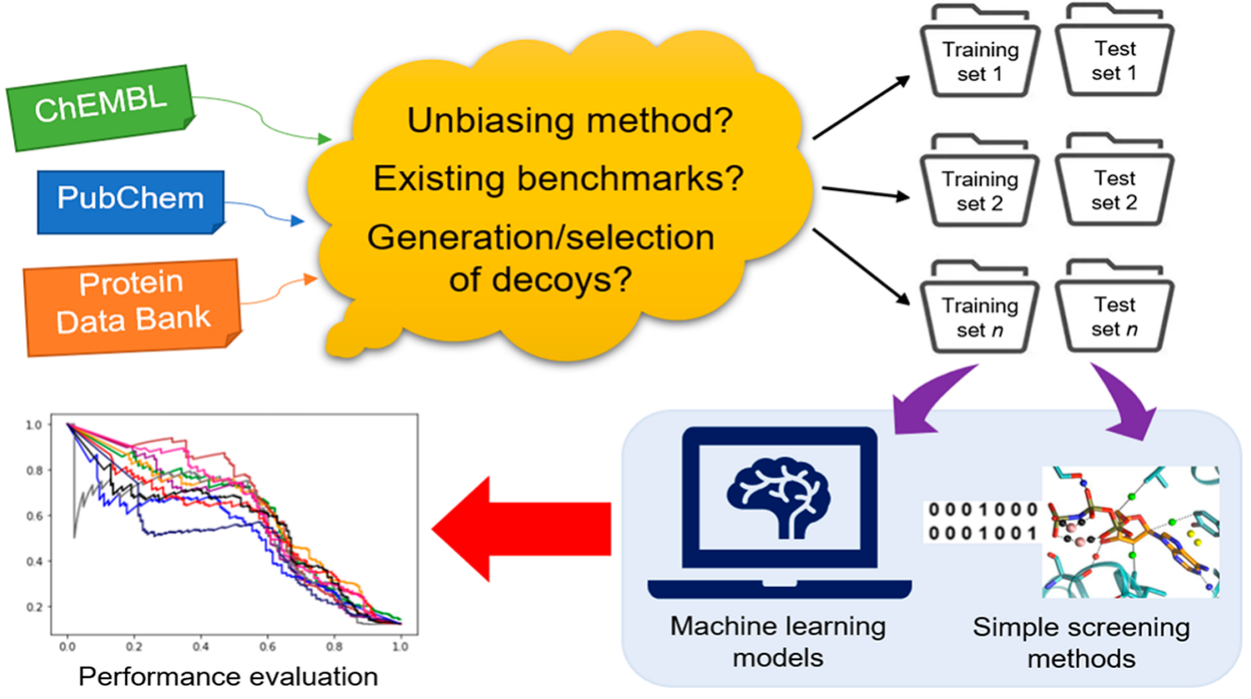

We discuss how data unbiasing and simple methods such as protein-ligand Interaction FingerPrint (IFP) can overestimate virtual screening performance. We also show that IFP is strongly outperformed by target-specific machine-learning scoring functions, which were not considered in a recent report concluding that simple methods were better than machine-learning scoring functions at virtual screening.

It is now well-established that machine learning (ML) scoring functions (SFs) excel at predicting the binding affinity of protein–ligand complexes from their crystal structures. This was originally shown by comparing the first version of RF-Score with 16 classical SFs,^1^ and this performance gap has been expanding ever since fueled by the growing availability of data and code to reproduce these results and build upon them.^2−7^ However, to excel at virtual screening as well, an ML SF needs to be trained on data sets with a large proportion of negative instances,^8,9^ typically assumed and called decoys. Using the same method to generate decoys, both for the training set and the test set, results in artificially improving virtual screening performance.^8^ Learning such decoy bias can be avoided by simply selecting decoys differently in each data set.^10^ The prospective yield of the model can also be overestimated by using test molecules that are easy to discriminate given the training set composition. Asymmetric Validation Embedding (AVE)^11^ benchmarks have been proposed to improve this situation,^11^ and simple models have been claimed to perform better than ML models.^12^ Here we assess the importance of comparing beyond AVE and just two generic ML SFs.

AVE is an innovative method that aims at designing unbiased data sets for ligand-based classification problems such as those in virtual screening. In particular, AVE has been used for training and evaluating ML SFs. Starting from a set of molecules labeled as either active or inactive for a given target, AVE generates a benchmark by partitioning this set into four subsets (training actives, training inactives, test actives, test inactives) such that the test molecules are not too similar to the training molecules, and the training actives/inactives are not too different from the test inactives/actives, according to their ECFP4 fingerprints.^13^ AVE was claimed to prevent molecular property prediction models, including ML SFs, from overfitting the benchmarks it generates.^11^

We select acetylcholinesterase (ACHE) and HMG-CoA reductase (HMGR) to illustrate the discussion. In another study, these targets were selected for reasons unrelated to the performance of the investigated SFs (two generic and six target-specific).^14^ As these targets obtained neither the best nor the worst performance on their corresponding DEKOIS2.0 test sets,^15^ we also select them for this study. Furthermore, these targets were cocrystallized in high resolution, with at least a ligand having the same bioactivity as those reported on PubChem^16^ and ChEMBL,^17^ and have never been assayed in any high-throughput screening campaign on PubChem (as of January 2023).

The experimental design comprised the following steps: retrieving experimental data (true actives and true inactives) of the two targets from PubChem and ChEMBL, downloading the benchmarking sets ACHE and HMGR from DEKOIS2.0,^15^ generating property-matched decoys with DeepCoy,^18^ a deep learning method for designing diverse and likely inactive molecules by tailoring their chemical features to those of an input active, from PubChem/ChEMBL true actives, and molecular docking with Smina v2019-10-15.^19^ Initial SMILES strings (PubChem/ChEMBL/DeepCoy) and sdf structures (DEKOIS2.0) of the ligands were converted into three-dimensional (3D) multi-mol2 files using Open Babel v2.3.1^20^ with the locations of hydrogen atoms being assigned at the physiological pH as usual. The PDB IDs 1EVE and 1HW8 were, respectively, used as target templates for ACHE and HMGR whenever DEKOIS2.0 ligands were involved (Table 1), whereas two other PDB structures 1E66 (ACHE) and 3CCW (HMGR), as proposed by DUD-E (https://www.dude.docking.org/targets) authors, were employed in other circumstances (i.e., for docking PubChem/ChEMBL/DeepCoy ligands), as we would like to use different receptor conformations to train and test our ML models. All target structures and their cocrystallized ligands were prepared with Chimera v1.15 (*Dock Prep* tool),^21^ according to parameters recommended by dos Santos et al. (2018).^22^ Docking was carried out within a search space of 27 000 Å^3^ (30 Å on each dimension) whose center was placed on the X-ray ligand of each protein; all other parameters were set following Smina recommendations.^19^

**Table 1:** Information on the Training-Test Data Splits Prepared for the Targets ACHE and HMGR

	ACHE data		HMGR data	
Split	DEKOIS2.0	AVE	DEKOIS2.0	AVE
***Training set***				
Source	PubChem/ChEMBL	PubChem/ChEMBL and DeepCoy	PubChem/ChEMBL	PubChem/ChEMBL and DeepCoy
Number of actives	166	125	113	110
Number of inactives	199	6520	923	6238
Nature of inactives	True inactives	True inactives and DeepCoy decoys	True inactives	True inactives and DeepCoy decoys
***Test set***				
Source	DEKOIS2.0	PubChem/ChEMBL and DeepCoy	DEKOIS2.0	PubChem/ChEMBL and DeepCoy
Number of actives	40	37	40	31
Number of inactives	1200	2170	1200	2077
Nature of inactives	Property-matched decoys from ZINC	True inactives and DeepCoy decoys	Property-matched decoys from ZINC	True inactives and DeepCoy decoys

Two different training-test partitions were prepared for each target, one with the ACHE/HMGR data set of DEKOIS2.0 as test set and the PubChem/ChEMBL data (excluding any molecules already in the test set) as training set for target-specific ML SFs. We call this split DEKOIS2.0, which was effectively made at random with training inactives being selected differently from test inactives to avoid decoy bias.^10^ The other partition, called AVE, was issued by the AVE script (training-to-test ratio = 3, other parameters set as default^11^), splitting the same population of ACHE/HMGR true actives and true inactives retrieved from PubChem/ChEMBL, plus DeepCoy-generated decoys property-matched to these true actives. These partitions are summarized in Table 1.

Five target-specific ML SFs were trained using the training sets of the aforementioned partitions. They used the following five algorithms: random forest (RF),^8,23^ extreme gradient boosting (XGB),^24,25^ support vector machine (SVM),^26,27^ artificial neural network (ANN),^28,29^ and deep neural network (DNN).^30,31^ Protein–Ligand extended connectivity (PLEC) fingerprints^32^ were used as features to describe ligand–receptor complexes after docking. Each of these ML SFs was then evaluated on the test set corresponding to its training set (hereinafter referred to as ACHE-DEKOIS2.0, ACHE-AVE, HMGR-DEKOIS2.0, and HMGR-AVE). Note that none of the training and test set pairs have any molecule in common by construction (in AVE splits, molecules in one set are, in addition, dissimilar to those in the other set). On the other hand, four generic SFs: Smina,^19^ IFP,^12^ CNN-Score,^33^ and RF-Score-VS v2^8^ were also tested on the same four test sets as those of the target-specific ones. Table 2 summarizes the performance of nine SFs mentioned above, in terms of area under the precision-recall curve (PR-AUC). The PR-AUC of each target-specific ML SF on each test set is the median value obtained after 10 training-test runs. We used PR-AUC as the performance metric, as it is more informative than ROC-AUC in strongly class-imbalanced test sets^34^ such as those in virtual screening.

**Table 2:** PR-AUCs of Nine SFs on Four Test Sets: ACHE-DEKOIS2.0, ACHE-AVE, HMGR-DEKOIS2.0, HMGR-AVE^a^

Test set	ACHE-DEKOIS2.0	ACHE-AVE	HMGR-DEKOIS2.0	HMGR-AVE
*Generic SF*				
Smina	0.195	0.083	0.080	0.020
IFP	0.167	0.021	0.125	0.084
CNN-Score	0.055	0.030	0.171	0.026
RF-Score-VS v2	0.083	0.050	0.763	0.034
*Target-specific ML SF*				
RF	0.518 [0.511, 0.528]	0.525 [0.524, 0.532]	0.968 [0.963, 0.972]	0.645 [0.641, 0.650]
XGB	0.165 [0.165, 0.165]	0.501 [0.501, 0.501]	0.886 [0.886, 0.886]	0.688 [0.688, 0.688]
SVM	0.566 [0.566, 0.566]	0.438 [0.438, 0.438]	0.961 [0.961, 0.961]	0.675 [0.675, 0.675]
ANN	0.094 [0.082, 0.114]	0.458 [0.452, 0.462]	0.946 [0.941, 0.947]	0.671 [0.667, 0.672]
DNN	0.091 [0.086, 0.127]	0.416 [0.392, 0.416]	0.791 [0.772, 0.849]	0.717 [0.682, 0.717]
*Expected at random*				
P/(P+N)	0.032	0.017	0.032	0.015

a Underlined are the cases where an AVE test set led to better virtual screening performance than the corresponding DEKOIS2.0 test set using the same SF. For target-specific ML SFs, the PR-AUCs outside square brackets are median values after 10 training-test runs, while those inside square brackets are the 1st (lower) and 3rd (upper) quartiles of the 10 runs. For each test set, the PR-AUC expected by a random-guessing model is P/(P+N), where P and N are the numbers of positives (actives) and negatives (inactives)^34^.

Table 2 shows that an SF performed better on an AVE test set than on the corresponding DEKOIS2.0 one in three of the 36 cases (8.3%). All of these cases involve the ACHE target and three target-specific ML SFs (XGB, ANN, DNN). The four generic SFs (two non-ML SFs and two ML SFs) gave better performance on DEKOIS2.0 test sets in all instances. Therefore, as expected, test sets by AVE are generally more challenging than their DEKOIS2.0 counterparts (Figure 1).

**Figure 1:**
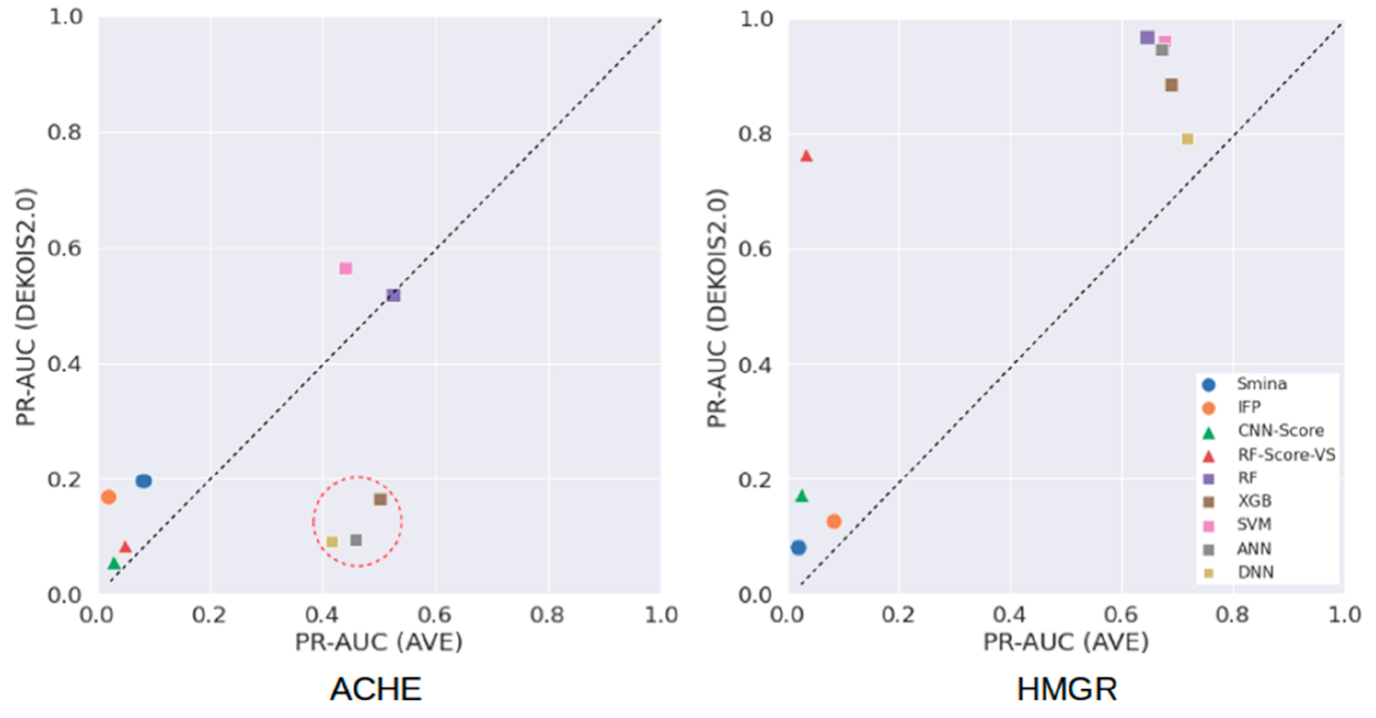
PR-AUCs of nine SFs on the two test sets (DEKOIS2.0 and AVE) of each target (ACHE, HMGR). The PR-AUC of each target-specific ML SF is the median value obtained after 10 training-test runs. Most SFs achieved higher PR-AUCs on DEKOIS2.0 benchmarks than on the corresponding AVE test sets. There are only three cases where an AVE-issued test set led to much better virtual screening performance by an SF than DEKOIS2.0: these cases are represented by three squares inside the red dotted circle in the ACHE plot and correspond to three target-specific ML SFs employing either XGB, ANN, or DNN as learning algorithm.

However, these three cases demonstrate that AVE benchmarks can sometimes be much easier than those generated effectively at random. For example, the bias of ACHE-DEKOIS2.0 in Table 2 is substantially larger than the bias of ACHE-AVE (0.387 vs 0.002), yet PR-AUCs for the XGB model are much larger when the ACHE-AVE partition is used (0.165 vs 0.501). This is due to the AVE bias not always being well-correlated with how challenging a data set is in practice, which could already be seen in the AVE study. Indeed, a careful inspection of that paper will identify data sets with practically no bias according to AVE but ROC-AUCs of up to 0.9 (e.g., some targets in Figure 11^11^).

We agree that AVE data splits are suited to compare ML and non-ML methods for virtual screening in a distribution-shift scenario. However, caution should be taken when one interprets AVE retrospective performance in absolute terms. For example, despite HMGR-AVE having a bias of just 0.008, the DNN model obtained a median PR-AUC as high as 0.717 with a 100% hit rate within the 21 highest-ranked molecules. While many studies achieve excellent prospective hit rates,^23,24,27,35−41^ almost never 100% is obtained, and it is safe to assume that there are many more unpublished prospective experiments due to not yielding any discovery. Therefore, we would certainly expect the prospective performance of these SFs to be considerably worse than that obtained with AVE splits. We do not recommend either to use AVE-trained models for prospective purposes, since its debiasing process has been found to reduce the information available to train a model, impairing its ability to generalize.^42^ Training with the most relevant data for the test set is a more promising approach.^43^

Regardless of partitioning data to have minimal AVE bias^11^ or differently generated decoys,^10^ it is clear that simple non-ML methods such as IFP are not the most suitable for structure-based virtual screening. This is particularly noticeable when using target-specific ML SFs (Figure 1). Furthermore, CNN-score strongly outperformed IFP in a PD-L1 benchmark.^44^ Markedly better performance than IFP has also been reported with ML SFs using the AVE-split HTS-based LIT-PCBA benchmark.^45^ None of these ML SFs were included in a study concluding that IFP was the best choice for structure-based virtual screening.^12^ Moreover, it is important to note that IFP is prone to overfitting retrospective benchmarks, in that one has to select the pose of a molecule bound to the target as the search template with knowledge of whether that 3D conformation is well-represented among test-set actives. As shown elsewhere,^46^ if this template is well-represented in the test set, then virtual screening performance will tend to be high (otherwise performance will be low), but this information is not available in prospective scenarios. Taken together, these counterexamples show the critical importance of broader comparisons to reach robust conclusions in this research topic.

## Accession Codes

The Python scripts and data sets related to this paper are freely available at https://github.com/vktrannguyen/AVE-vs-DEKOIS.
